# Therapeutic Applications of Rosmarinic Acid in Cancer-Chemotherapy-Associated Resistance and Toxicity

**DOI:** 10.3390/biom14070867

**Published:** 2024-07-19

**Authors:** Cecilia Villegas, Nicole Cortez, Ayorinde Victor Ogundele, Viviana Burgos, Paulo Celso Pardi, Jaime R. Cabrera-Pardo, Cristian Paz

**Affiliations:** 1 Laboratory of Natural Products & Drug Discovery, Center CEBIM, Department of Basic Sciences, Faculty of Medicine, Universidad de La Frontera, Temuco 4780000, Chile; c.villegas04@ufromail.cl (C.V.); n.cortezsalvo01@gmail.com (N.C.);; 2 Department of Chemistry and Industrial Chemistry, Kwara State University, Malete 1530, Nigeria; 3 Departamento de Ciencias Biológicas y Químicas, Facultad de Recursos Naturales, Universidad Católica de Temuco, Rudecindo Ortega, Temuco 4780000, Chile; vburgos@uct.cl; 4 Centro Universitario ENIAC, Guarulhos 07012-030, SP, Brazil; paulo.pardi@gmail.com; 5 Laboratorio de Química Aplicada y Sustentable, Departamento de Química, Facultad de Ciencias, Universidad de Tarapacá, Arica 1000000, Chile; jacabrera777@gmail.com

**Keywords:** rosmarinic acid, chemotherapy adjuvant, cancer resistance, chemoprotection

## Abstract

Chemotherapeutic drugs and radiotherapy are fundamental treatments to combat cancer, but, often, the doses in these treatments are restricted by their non-selective toxicities, which affect healthy tissues surrounding tumors. On the other hand, drug resistance is recognized as the main cause of chemotherapeutic treatment failure. Rosmarinic acid (RA) is a polyphenol of the phenylpropanoid family that is widely distributed in plants and vegetables, including medicinal aromatic herbs, consumption of which has demonstrated beneficial activities as antioxidants and anti-inflammatories and reduced the risks of cancers. Recently, several studies have shown that RA is able to reverse cancer resistance to first-line chemotherapeutics, as well as play a protective role against toxicity induced by chemotherapy and radiotherapy, mainly due to its scavenger capacity. This review compiles information from 56 articles from Google Scholar, PubMed, and ClinicalTrials.gov aimed at addressing the role of RA as a complementary therapy in cancer treatment.

## 1. Introduction

Drug resistance is recognized as the main cause of chemotherapeutic treatment failure in almost all human tumors and is highly dependent on the type of cancer, stage, and drug administered, as well as the comorbidities of the patient [1]. Cancer cells can develop resistance to chemotherapy drugs in two ways: intrinsic resistance, which exists from the start, meaning tumors are never affected by the treatment, and acquired resistance, which develops after some initial success with chemotherapy. In this case, a sub-population of cancer cells becomes resistant, allowing the tumor to regrow and become uncontrollable [2].

In cancer, combination therapy with another antineoplastic agent allows for both to act in concert to destroy as many cells as possible and reduce the probability that the cancer will become resistant to a particular drug [3]. For a long time, it has been suggested to combine natural products with traditional treatments [4] because these natural products can act on several molecular targets and redundant signaling pathways in cancer cells, hindering their growth and survival [5]. According to a review conducted by Sotiropoulou in 2014, about 30 natural molecules from different families have been identified as potential candidates against different types of cancer stem cells (CSCs) by reversing chemoresistance, of which retinoic acid, quercetin, mithramycin, and curcumin can be highlighted because, in addition to the results observed in vitro, there is evidence in clinical trials [6]. Wang et al. additionally reported 14 natural compounds with in vitro anti-resistance activities through the inhibition of drug transporters, cellular detoxification capacities, or increased sensitivities to apoptosis in different cell models resistant to conventional antineoplastics [7]. Later, in 2016, the roles of 12 polyphenols in reversing resistance by modulating the expressions of p53, P-gp, breast cancer resistance protein (BCRP/ABCG2), mitogen-activated protein kinase (MAPK), and nuclear factor kappa-light-chain-enhancer of activated B cell (NF-κB) signaling pathways [8] were investigated, and, finally, a systematic review, including 104 publications with preclinical data on the sensitization of tumor cells to chemotherapy by natural products, reported that phenolic derivatives (26.9%) and flavonoids (17.3%) are the main compound chemosensitizers, highlighting compounds such as curcumin, resveratrol, and epigallocatechin-3-gallate, which were used in combination therapy [9].

Prior to the publication of these reviews on chemoresistance, the potential of rosmarinic acid (RA) had not been explored. However, in recent years, evidence has emerged demonstrating the ability of RA to act as a chemosensitizer in vitro by regulating specific cellular pathways; RA may directly or indirectly enhance the efficacy of established anticancer drugs, resulting in improved treatment outcomes [10,11,12,13]. Several studies at the cellular level have examined the beneficial properties of RA, evidencing its potential as an antioxidant, antibacterial, antiviral, analgesic, anti-inflammatory, antidiabetic, hepatoprotective, cardioprotective, neuroprotective, and anticarcinogenic compound, proven in various types of malignancies, including colorectal, pancreatic, breast, lung, ovarian, and melanoma, among others [14].

**Figure 1:**
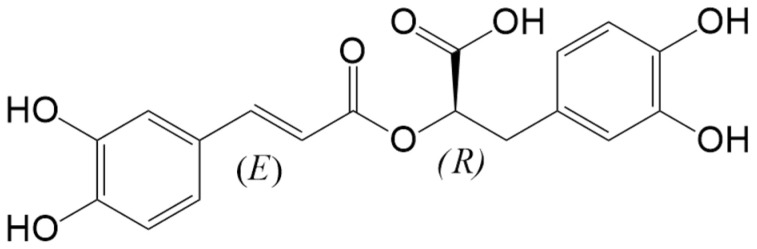
Chemical structure of rosmarinic acid (RA).

RA is a phenolic compound, an ester of caffeic acid and 3,4-dihydroxyphenylacetic acid (Figure 1), which gives rise to a molecule with pro-oxidant and antioxidant capacities [15]; the latter is supported by its ability to scavenge H_2_O_2_ and scavenge free radicals because of the presence of two catechol moieties, which provide the appropriate polarity for RA to penetrate lipid bilayers and protect them against oxidation without altering their structures [16]. The capacity of RA to directly counteract reactive oxygen species, owing to its chemical structure, combined with its potential to enhance the cell’s antioxidant defenses, plays a pivotal role in mitigating oxidative damage to non-tumor tissues. Conversely, within tumor tissue, RA exhibits interactions with specific proteins known to be dysregulated (Table 1), thereby augmenting the efficacy of chemotherapy drugs against the chemoresistance mechanisms inherent in these cells [17,18,19,20].

**Figure 2:**
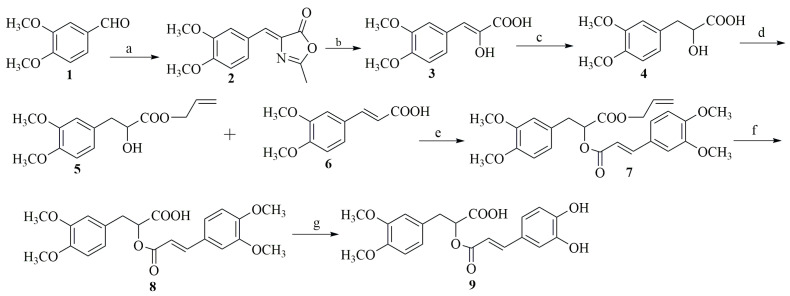
Chemical synthesis of RA. a = NaOAc, Ac_2_O, AcHNCH_2_COOH, 10 h, 110 °C, b = 3 mol/L HCl, 10 h, 90 °C, c = NaBH_4_, Ph = 10, from 0 °C to r.t., d = allyl alcohol, p-TsOH, Toluene, 12 h, refluxalcohol, p-TsOH, Toluene, 12 h, reflux, e = DCC, DMAP, CH_2_Cl_2_, r.t., f = Pd(PPh_3_)_4_, morpholine, THF, r.t., g = Me_3_SiI, quinoline, 4 h, 180 °C.

The first total synthesis of RA was accomplished by Albrecht in 1991 [17]. They employed piperonyl chloride as the starting material, synthesizing RA through a nine-step reaction sequence. In 1996, Theophil et al. used piperonal as the raw material to obtain rosmarinic acid and its derivatives via different synthetic routes, achieving an overall yield of approximately 5% [18]. Yuan, in 2011, developed a more efficient synthesis method. Using veratraldehyde as the starting material, they achieved a 30% yield after a seven-step process involving the Erlenmeyer reaction, hydrolysis ring opening, reduction, protection, condensation, and deprotection [19] (Figure 2).

Chemotherapy is associated with significant toxicity in other tissues, negatively impacting the patient’s quality of life. These effects can be reduced using chemoprotectants, which are molecules that diminish these effects without affecting the efficacy of the main drug. Over the years, synthetic molecules have been developed for this purpose, including amifostine, aprepitant, dexrazoxane, filgrastim, sargramostim, mesna, oprelvekin, palifermin, and recombinant human erythropoietin [20]. Numerous phytopharmacological derivatives, such as flavonoids and polyphenols, appear promising in combination with chemotherapy, as they can reduce the side effects of chemotherapy and enhance the efficacy of anticancer drugs. However, the use of natural supplements in conjunction with chemotherapy remains under debate [21,22,23].

Recent findings indicate that RA could be an alternative in chemoprotection due to its antioxidant and anti-inflammatory capabilities through regulation of the NF-κB pathway [24]. In this review, we discuss the biological functions and therapeutic applications of RA, highlighting its efficacy as an adjuvant for the treatment of chemotherapy-associated resistance and toxicity. We also summarize its phytochemical aspects.

## 2. Methodology

A comprehensive literature search was conducted in PubMed/MEDLINE using the search term “cancer AND chemoresistance”, yielding a total of 16,386 articles. The search was then refined by applying the filter “cancer and rosmarinic acid” NOT “chemoprevention”, resulting in 247 articles. Further refinement involved an advanced search combining “rosmarinic acid” with terms such as “chemoresistance”, “resistance”, “chemosensitization”, “reversal of resistance”, “lipid peroxidation”, and “chemoprotective” or “chemotherapy toxicity protector”, resulting in 164 articles. A manual review of these articles excluded non-cancer-related and pre-2010 publications, leaving 56 articles for inclusion in the discussion. Additionally, an extensive search was conducted in Google Scholar, associating “Rosmarinic acid” with specific drugs used in oncological chemotherapy. The bibliographic selection prioritized original research to ensure the quality of the review article.

## 3. Phytochemical Aspects of Rosmarinic Acid

Rosmarinic Acid (C_18_H_16_O_8_) is synthesized through the esterification of caffeic acid and 3-(3,4-dihydroxyphenyl) lactic acid. Its systematic name is (*R*)-α-[[3-(3,4-dihydroxyphenyl)-1-oxo-2*E*-propenyl]oxy]-3,4-dihydroxybenzenepropanoic acid, and it features a chiral center that forms *S* (−) and *R* (+) enantiomers. This compound was first isolated in 1958 by Italian chemists Scarpati and Oriente as a pure substance. It is named after the rosemary plant (*Rosmarinus officinalis* L.), from which it was initially extracted, and is recognized as an ester of 3,4-dihydroxyphenyllactic acid [25]. Before its structure was elucidated, RA and many of its derivatives were identified as “Labiatengerbstoffe”, tannin-like substances found in plants of the Lamiaceae family. It was initially thought that the tannin-like compound extracted from *Melissa officinalis* included caffeic acid and could not be categorized as a gallotannin, ellagitannin, or condensed catechin. The chemical synthesis of RA was achieved in 1991 by Albrecht, leading to the creation of numerous derivatives and stereoisomers since then [25]. Caffeic acid acts as a fundamental building block in a variety of secondary metabolites, which range from simple monomers to more complex compounds and their oligomers. Among the therapeutically significant compounds derived from caffeic acid are its trimers and tetramers. Caffeic acid in its monomeric forms typically appears as caffeic acid itself or as 3-(3,4-dihydroxyphenyl) lactic acid. Other monomeric derivatives include ferulic acid, isoferulic acid, and chlorogenic acid. Notably, while chlorogenic acid is common in fruits, it is rarely found in the Lamiaceae family, where it is instead replaced by RA [26]. Rosmarinic acid is one of the most common caffeic acid dimers found in plants and is well-known for its impressive biological properties, especially its role in anti-cancer therapies [27].

In plant sources, multiple derivatives of RA have been identified, featuring one or two RA units linked with other aromatic groups. Notably, lithospermic acid and lithospermic acid B are among the most common of these derivatives [28].

## 4. Occurrence and Distribution of Rosmarinic Acid in Nature

Rosmarinic Acid has been identified or isolated from a total of 162 plant species spanning 26 plant families [29]. Notably, the Lamiaceae family encompasses the highest number of plants, with 104 species containing RA. Salvia is the most abundant genus containing RA. Although RA is primarily found in extracts of *Basilicum polystachyon*, it is also present in various other plants, including *Thymus mastichina* and species within the Lamiaceae family, specifically in the Agastache genus [30]. RA is prevalent in the Labiatae family, predominantly located in the vacuoles and cytoplasm as an anion in *Mentha spicata*, exhibiting limited membrane diffusion [29]. Among Mentha species, particularly *M. spicata*, the highest RA content is observed [31]. Additionally, this phenolic acid accumulates in taxa like Choranthaceae and Blechnaceae, as well as several marine hydrophilus angiosperms. Rosmarinic acid is distributed among various plant categories, encompassing primitive and advanced terrestrial plants, along with monocotyledonous and eudicotyledonous species [32]. Though extensively dispersed throughout the plant realm, it acts as a notable chemical marker mainly for the Lamiaceae family in terms of chemotaxonomy. The presence of RA offers taxonomic insights, particularly at the subfamily level [29]. According to the European and Mediterranean Plant Protection Organization database, among the 104 Lamiaceae plants, 93 belong to Nepetoideae and 10 to Lamioideae (“European and Mediterranean Plant Protection Organization”, n.d.). RA serves as a characteristic natural product distinguishing Nepetoideae from other subfamilies in Lamiaceae. In relation to the content of RA in plants, a comparative analysis suggests concentrations reaching as high as 58.5 mg/g of dried plant matter.

### 4.1. Biosynthesis of Rosmarinic Acid

**Figure 3:**
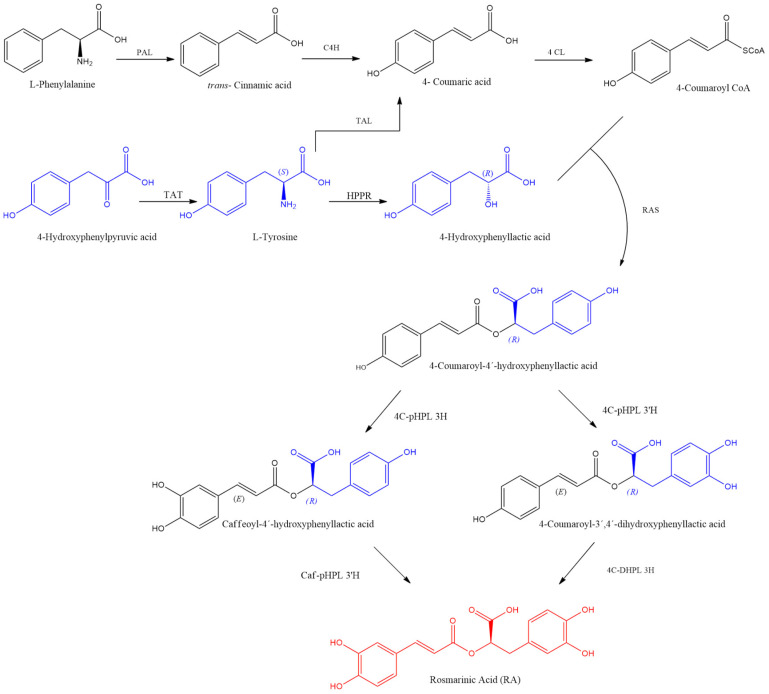
Biosynthetic pathways for the formation of RA, marked in red. The involved enzymes are abbreviated: PAL = phenylalanine ammonia lyase; C4H = cinnamate 4-hydroxilase; 4CL = 4-coumaric acid CoA-ligase; TAT = tyrosine aminotransferase; HPPR = hydroxyphenylpyruvate reductase; RAS = rosmarinic acid synthase, hydroxycinnamoyl-CoA:hydroxyphenyllactate hydroxycinnamoyltransferase; 4C-pHPL 3H, 4C-pHPL 3′H = 4-coumaroyl-4′-hydroxyphenyllactate 3/3′-hydroxylases; Caf-pHPL 3′H = caffeoyl-4′-hydroxyphenyllactate 3′-hydroxylase; 4C-DHPL 3H = 4-coumaroyl-3′,4′-dihydroxyphenyllactate 3-hydroxylase.

Rosmarinic acid (RA) is produced via phenylpropanoid metabolism, using the amino acids phenylalanine or tyrosine as a precursor. Knockout mutations and RNAi-mediated suppression in Arabidopsis and other model plants have facilitated the identification and functional characterization of genes and enzymes involved in the pathway [33,34], as schematized in Figure 3.

The biosynthesis of RA has been studied in *C. blumei* [26] and reviewed by Kim, et al. [35]. RA is synthesized with the participation of two precursors; L-phenylalanine is transformed to 4-coumaroyl CoA, and L-tyrosine is transformed to 4-hydroxyphenyllactic acid. The coupling of these compounds occurs through the enzyme rosmarinic acid synthase (RAS), with the release of coenzyme A. Esterification occurs at the alpha position to the carboxylic acid of the 4-hydroxyphenyllactic acid moiety, giving (*R*) configuration to the new center, forming the compound 4-coumaroyl-4′-hydroxyphenyllactic acid (4C-pHPL). Further, the *meta*-hydroxyl groups (3 and 3′) are introduced by aromatic meta-hydroxylases through reactions of the cytochrome P450-dependent monooxygenase from the CYP98A family [26].

### 4.2. Absorption, Distribution, and Metabolism

The metabolism and bioavailability of RA have been studied in animal models, revealing rapid absorption in the stomach and intestine [36,37]. The main route of excretion is through urine, occurring approximately 6 to 8 h after ingestion. RA undergoes transformations both by tissues and by microbiota, yielding sulfate and glucuronide derivative molecules as the main derivatives. In studies with rats deprived of food for 12 h, followed by administration of RA at a concentration of 50 mg/kg body weight, blood and urine samples were collected from 0 to 8 h and from 8 to 18 h post-administration. Monomethylated RA was detected within the first hour, while by 8 h, m-coumaric acid predominated. These compounds were primarily present in conjugated forms such as glucuronides or sulfates. Urine was the primary route of RA excretion, with 83% of the total metabolites excreted during the period from 8 to 18 h after RA administration [38].

The metabolism of RA in healthy humans was studied following ingestion of a single dose of *Perilla frutescens* extract, a plant rich in RA. In urine, the detected compounds included RA, methylated RA, caffeic acid, ferulic acid, and traces of m-coumaric acid. In plasma, RA, methylated RA, and ferulic acid were identified. In both urine and plasma, these compounds were present predominantly as glucuronide and/or sulfated conjugated forms [39].

## 5. Rosmarinic Acid as a Food Additive

Rosmarinic acid, characterized by its hydroxyl groups, reveals very strong antioxidant activity and is therefore interesting as a functional food ingredient. In this regard, studies conducted have identified increasing antioxidant activity from RA compared to antioxidants like sulfoxide. In recent years, several scientific investigations have demonstrated that lipid oxidation, particularly in processes like deep frying during food preparation, results in the formation of potentially hazardous compounds. These substances encompass oxidized lipids, trans fats, sterol byproducts, acrylamide, and heterocyclic substances. This may result in both deterioration and negative modifications in the quality of food products, along with the production of detrimental substances. One such substance is malondialdehyde (MDA), which has the potential to initiate various inflammatory responses in humans, including carcinogenesis [40]. The process of oxidation involves a series of sequential reactions involving free radicals. These reactions initiate when food or its constituents, including fatty acids and proteins, are exposed to factors such as heat, light, ionizing radiation, metalloprotein catalysis, or metal ions [41]. Therefore, various techniques have been explored to extend the shelf life of food, prevent oxidation, and reduce the formation of unwanted byproducts. One frequently used approach is to add synthetic antioxidants such as butylated hydroxyanisole (BHA), butylated hydroxytoluene (BHT), or tertiary butyl hydroxyquinone (TBHQ), which is a direct and commonly practiced method [42]. Presently, there is growing consumer apprehension regarding the safety of synthetic antioxidants, prompting a rise in the utilization of natural polyphenols instead. Among these, RA and extracts containing RA, such as rosemary extract, stand out as the most prevalent natural antioxidants integrated into various food items [41]. Rosemary extract has been approved in China at levels ranging from 300 to 700 mg/L, and in the European Union, from 30 to 250 mg/L, depending on the food category. The antioxidant properties of the extract mainly come from non-polar phenolic diterpenes such as carnosic acid, carnosol, and rosmanol. Additionally, China has set limits on the use of TBHQ at 0.2 g/kg, while its usage is progressively decreasing in Japan, Canada, and Europe [40]. In this aspect, both RA and rosemary extract, typically containing around 4–4.5% RA, have undergone extensive analysis in various experimental settings and real-world food scenarios. These investigations have encompassed a range of food products, including edible oils, processed meats, dairy goods, and beverages. Their effectiveness as antioxidants and their ability to prevent food oxidation during storage through co-pigmentation effects have been thoroughly explored [43,44,45,46]. Moreover, their stability within the specific food systems under investigation has been evaluated. Additionally, attention has been given to the formation of undesirable compounds resulting from RA degradation and its interaction with amino acids, which may compromise meat quality and pose risks to human health [45].

Researchers are intrigued by the potential of omega-3 essential fatty acids to improve various physiological aspects in humans, including triglyceride levels, blood pressure regulation, and cancer prevention. Within a simulated system containing palmitic acid methyl ester (C16:0), stearic acid methyl ester (C18:0), oleic acid methyl ester (C18:1), linoleic acid methyl ester (C18:2), and linolenic acid methyl ester (C18:3), among 22 polyphenols examined, RA, myricetin, and carnosic acids showed the highest effectiveness in preventing omega-3 oil oxidation. This significant antioxidant activity is attributed to the catechol-type ring structure, conjugated double bonds, and alkyl substituents found in the phenol rings of these polyphenols. These structural characteristics contribute to low bond dissociation enthalpies, indicating the antioxidant’s ability to donate electrons, enabling RA to scavenge four radicals per molecule. This establishes RA as a superior natural substitute with greater antioxidant efficacy when contrasted with α-tocopherol, BHA, BHT, and TBHQ [43]. In the high-temperature frying of foods (155–190 °C), like French fries, RA and RA-derived antioxidants exhibited superior efficacy in thwarting soybean oil oxidation compared to TBHQ, as evaluated through color preservation and sensory assessments [40]. A mixture of spices containing a high level of RA was employed to inhibit lipid oxidation and the formation of MDA while cooking hamburger meat. Adding the spice blend at a ratio of 11.3 g per burger led to a reduction of 71% in MDA formation compared to burgers prepared without the spice blend. Furthermore, individuals who consumed the burgers with the spice blend showed a 49% decline in urinary MDA levels [44]. Incorporating *L. vera* extract, abundant in RA (95.3 mg/g dry extract), led to reduced lipid oxidation and preservation of tocopherol levels in cooked meat over a 10-day storage period. These results further support the effectiveness of RA as a natural antioxidant [47].

RA has been extensively studied not only for its antioxidant properties but also for its ability to stabilize and improve the color of beverages like juices and wines. These beverages often contain unstable natural colorants like anthocyanins and carotene [48]. Compared to other phenols such as caffeic acid and danshensu, RA has shown superior color-stabilizing properties in wine. The hyperchromic effects of RA, which increase color intensity, and its bathochromic effect, which shifts the absorption wavelength to longer wavelengths, varied between 2 and 161% and 7 and 19 nm, respectively, at ratios ranging from 1:10 to 1:60. In contrast, caffeic acid exhibited hyperchromic effects ranging from 2 to 82% at equivalent molar concentrations. Moreover, higher molar proportions of RA resulted in more intense coloration, producing deeper and more vibrant blue tones [46].

## 6. Role of Rosmarinic Acid in Reverse Cancer Chemoresistance

Chemoresistance is a complex phenomenon involving multiple interrelated or independent mechanisms that contribute to disease relapse and metastasis in cancer [49,50]. Malignant tumors maintain active proliferative signaling pathways, facilitated by cancer stem cells (CSCs), a subpopulation capable of self-renewal, initiating metastasis, undergoing epithelial–mesenchymal transition (EMT), and causing chemoresistance. This resistance is often mediated by overexpression of several ATP-binding cassette (ABC) transporters that efflux chemotherapeutic agents from the cytosol to the extracellular space, along with mechanisms involving active DNA repair and resistance to apoptosis [51]. Studies indicate that combining natural products with chemotherapeutic drugs can enhance cytotoxic effects by modulating alternative pathways to induce apoptosis or by inhibiting cellular efflux transporters. [9]. This approach not only increases therapy efficacy but also potentially reduces chemotherapeutic doses, thereby mitigating toxicity.

There are currently eight recognized mechanisms underlying chemoresistance [52]: tumor heterogeneity; inactivation of drugs (cytochrome P450, glutathione-S-transferase (GST), uridine diphospho-glucuronosyltransferase (UGT) superfamily); overexpression of drug targets (epidermal growth factor receptor (EGFR) and its downstream signaling targets); efflux pump overexpression (MDR or P-gp, MRP1, ABCG2); DNA damage repair; evasion of cell death mediated by Bcl-2, Akt, NF-κB and STAT; epithelial–mesenchymal transition (EMT) favored by overexpression of transforming growth factor β (TGF-β), focal adhesion kinase (FAK) and vascular endothelial growth factor (VEGF); and epigenetic changes [53]. Understanding these mechanisms has facilitated the development of strategies aimed at enhancing conventional drug therapies to overcome these barriers [54].

**Figure 4:**
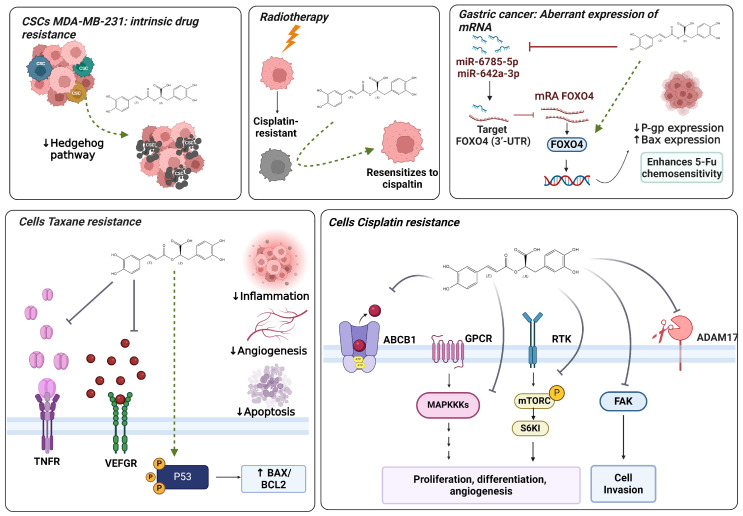
Effects of rosmarinic acid on molecular pathways mediating chemotherapy resistance in cancer.

RA, well known for its antioxidant properties, has recently been identified as a potential pro-oxidant in the presence of superoxide anions. Studies indicate that RA can facilitate the reduction of Cu (II) to Cu (I) and Fe (III) to Fe (II) leading to Fenton-type reactions that generate reactive hydroxyl radicals (HO˙). These radicals are implicated in DNA damage and induction of apoptosis in cancer cells. Moreover, phytochemicals like RA exert antitumor effects through various mechanisms, including selective elimination of rapidly dividing cells, modulation of abnormal molecular factors, and regulation of cell growth factors. Importantly, RA interacts with specific dysregulated proteins in cancer cells (Figure 4), and its combined use with chemotherapeutic agents may enhance their effectiveness, potentially mitigating chemoresistance mechanisms in tumor cells.

In the context of reverse cancer chemoresistance, RA has demonstrated potential in controlling breast cancer stem cells (CSCs). This effect was studied using cells with stem cell characteristics (CD44/CD24-/low) isolated from the human breast cancer cell line MDA-MB-231 through fluorescence-based cell sorting. It was found that RA inhibits stem-like breast cancer cells by targeting the hedgehog signaling pathway and modulating the Bcl-2/Bax ratio at concentrations of 270 and 810 μM [55]. This finding is crucial because CSCs, known for their intrinsic drug resistance, are a significant contributor to chemo- and radiotherapy resistance in cancer [56].

Additionally, RA has shown efficacy in reversing multidrug resistance (MDR) in various cancer cell lines. It has been observed to downregulate P-glycoprotein (P-gp) expression and decrease MDR1 gene transcription, thereby reversing MDR. In vitro studies have demonstrated RA’s ability to reverse MDR in doxorubicin-resistant SGC7901 cells, increasing the intracellular accumulation of doxorubicin and Rh123 (Figure 4). Similarly, in cisplatin-resistant A549 cells (A549/CDDP, lung cancer), RA supplementation significantly reduced MDR1 expression levels, associated with increased apoptosis. These results suggest that RA could be effective in overcoming MDR-mediated resistance in non-small cell lung carcinoma (NSCLC) [10].

In clinical practice, cisplatin is a crucial chemotherapeutic agent used for treating various solid tumors, including microcytic and non-small cell lung cancers, head and neck cancers, cervical cancer, testicular cancer, ovarian cancer, bladder cancer, and stomach cancer. In ovarian cancer, carboplatin, cisplatin, and paclitaxel form the standard regimen, yet the high relapse rate, which can affect up to 50% of patients, is largely attributed to chemoresistance [57]. Similarly, gastric cancer exhibits high relapse rates, with over 50% of operated patients experiencing local recurrence, and the 5-year survival rate in metastatic cases remains less than 10% [58]. Unfortunately, most patients with advanced gastric cancer eventually develop resistance to 5-fluorouracil (5-FU) [59], which is commonly used alone or in combination with cisplatin or epirubicin as per the the National Comprehensive Cancer Network^®^ (NCCN) guidelines [60].

Enhancement in the cellular response to platinum derivatives is also achieved through modulation of specific proteins involved in cell signaling, one of them being ADAM17, a disintegrin and metalloproteinase highly expressed in almost all cancers whose overexpression significantly decreases the effect of chemotherapy on tumor growth. Downregulation of ADAM17 is associated with increased apoptosis and reversal of oxaliplatin resistance in colorectal cancer [61].

RA has been reported to modulate the ADAM17/EGFR/AKT/GSK3β signaling axis in A375 melanoma cells, potentially enhancing synergy with cisplatin [62]. Although direct investigation into RA’s role in reversing ADAM17-associated chemoresistance was not found in this review, its ability to modulate this protein suggests a possible role in the process. However, further studies are required to fully understand RA’s impact on ADAM17-mediated chemoresistance.

ADAM17 has also been proposed as a target for radiosensitization in non-small cell lung cancer, suggesting that repurposing ADAM17 inhibitors could serve as short-term adjuvants to improve outcomes in radiotherapy settings [63].

The study by Liao et al. demonstrated that RA can reverse the resistance of non-small cell lung cancer (NSCLC) to cisplatin by activating the MAPK signaling pathway, specifically JNK/c-Jun. They observed that combined treatment with RA and cisplatin significantly increased the sensitivity of NSCLC cells to cisplatin, with a combination index (CI) indicating a synergistic effect (CI < 1). In vivo analysis further showed that RA and cisplatin combination therapy more effectively inhibited tumor growth compared to monotherapy in a xenograft model of NSCLC [10].

Additionally, research focusing on RA-rich extracts supports these findings. An extract from *Glechoma hederacea* (ground ivy), when administered alongside cisplatin, improved the inhibition of metastatic renal cancer cells (RCC 786-O). This extract induced cell cycle arrest in the G2/M phase and inhibited cell invasion at concentrations of 25–50 μM, achieved through negative regulation of FAK (focal adhesion kinase) [64]. FAK is known to be overexpressed in various cancers and plays a role in promoting metastasis and cancer progression.

RAME, a derivative of RA, has shown promising potential in overcoming resistance in gynecological cancers by targeting specific molecular pathways. For instance, RAME interacts with pathways involving the transcription factor Forkhead box M1 (FOXM1), which is associated with proliferation and regulates genes controlling various phases of the cell cycle (G1/S, S, G2/M, and M phases) [65]. In studies conducted by Lim et al., treatment of ovarian cancer cells with RAME reduced the mRNA expression of FOXM1 target genes. Combined treatment of RAME with cisplatin sensitized cisplatin-resistant ovarian cancer cells, enhancing their response to chemotherapy [11].

Furthermore, RAME has been investigated for its effects on the mTOR/S6K1 axis, a pathway commonly activated in cervical cancer and targeted in therapeutic strategies. RAME was found to inhibit mTOR-mediated S6K1 activation by disrupting the interaction between S6K1 and mTOR. This action enhances the antitumor effects of cisplatin in cisplatin-resistant cervical cancer cells by inducing autophagy and apoptosisautophagy and apoptosis [66].

The molecular mechanisms through which RA and its derivatives, like RAME acton chemoresistant cells, act on cisplatin and other antineoplastic agents are illustrated in Figure 4.

RA has demonstrated effectiveness in enhancing chemosensitivity to 5-FU, a commonly used chemotherapy agent for gastrointestinal cancers. In studies by Yu et al., RA treatment significantly increased the rate of apoptosis in SGC7901/5-Fu-resistant cells. This effect was attributed to RA’s ability to downregulate microRNAs miR-6785-5p and miR-642a-3p, which normally suppress the expression of the tumor suppressor FOXO4. By upregulating FOXO4 expression, RA restored the sensitivity of cells to 5-FU, thereby enhancing its efficacy as a treatment option [13].

While chemotherapy, radiotherapy, and surgery remain the primary treatments for gastrointestinal cancers, immunotherapy targeting specific antigens has emerged as a viable option for advanced stages. MUC1, a molecule implicated in tumorigenesis, inhibition of cell death, and promotion of metastasis [67], has been a focus of interest. Numerous anti-MUC1 antibodies are currently undergoing clinical trials or are being studied in preclinical and experimental settings [68]. In vitro studies have shown that combining anti-MUC1 therapy with RA is more effective than monotherapy, suggesting a promising new strategy in the treatment of gastric cancer [69].

The ability of RA to enhance the effectiveness of taxanes like docetaxel and paclitaxel has been investigated, demonstrating significant synergistic effects in cancer treatment. In MDA-MB-231 breast cancer cells, the combined treatment of RA with docetaxel showed a strong synergistic effect, increasing the antiproliferative properties of docetaxel by 70 ± 2.4% (*p* < 0.05) [70]. While this study did not directly assess the reversal of resistance, the synergistic action suggests potential benefits, such as reducing the required dose of docetaxel, which could indirectly delay the development of resistance and improve treatment response.

In vivo studies using an Ehrlich solid carcinoma (breast cancer) model further supported these findings. Mice treated with RA in combination with paclitaxel exhibited the most significant decrease in tumor weight (*p* < 0.001) compared to monotherapy. This combination therapy was associated with lower levels of tumor necrosis factor alpha (TNF-α) and vascular endothelial growth factor (VEGF), increased levels of P53 and caspase 3, and a shift in the Bcl2/Bax ratio favoring apoptosis. These effects collectively reduced inflammation and angiogenesis while enhancing apoptosis, leading to improved treatment outcomes [71].

Table 1 summarizes the findings from these investigations, including the doses of RA used in each study, highlighting RA’s potential as a supportive therapy in combination with taxanes for cancer treatment.

**Table 1:** Rosmarinic acid dose applied in preclinical studies associated with the reversal of chemoresistance to conventional drugs in cancer since 2015.

Cancer Type: Model	Treatment Conditions	Effect in Molecular Pathway	Potential Clinical Effects	References
Non-small cell lung cancer: A549 cells and A549DDP (cisplatin-resistant) In vivo: Nude female BALB/c-nu/nu mice	In vitro: RA (5–10 µg/mL) + cisplatin (IC_50_) for 48 h. In vivo: RA (10 mg/kg/day, IP*), and cisplatin once every 5 days	Downegulation of P-gp. JNK/c-Jun activation (MAPK signaling pathway)	Reversed clinical effects of multidrug resistance	[10]
Gastric cancer: SGC7901/Adr cells (Adriamycin-resistant)	RA (2.4 and 12 μM) + Adriamycin (IC_50_) for 48 h	Decreasing MDR1 gene transcription	Reversed multidrug resistance	[12]
Renal cancer: RCC 786-O cell	RA (25–200 µM) + cisplatin (5 µM) for 48 h	Inhibition of FAK posphorylation	Enhanced cisplatin potency	[64]
Malignant melanoma: A375 cells	RA (50–200 μg/mL) pre-treatment for 24 h, followed by cisplatin (8 μM) co-treatment for another 24 h	Downregulation of ADAM17/EGFR/AKT/GSK3β axis	Enhanced cisplatin potency	[62]
Solid Ehrlich Carcinoma: Female Swiss albino mice	RA (100 mg/kg/day/orally) daily + paclitaxel (10 mg/kg, IP) three times weekly	NF-κB, p53 and caspase-3 pathway modulation	Potentiated the therapeutic effect of Paclitaxel	[71]
Gastric cancer: SGC7901/5-Fu cells (5-Fu resistant)	RA (15 μg/mL) + 5-Fu (50 μg/mL)	Upregulation of FOXO4 expression	Reversal of 5-Fu Chemoresistance	[13]
Gastric cancer: AGS cells	RA (100–200 μM) + anti-MUC1 (5 μg/mL) of for 24 h	Downregulation of MUC1 expression and mRNA level of BAX, Bad, and caspase 9	Enhanced anti-MUC1 potency	[69]
Breast cancer: MDA-MB231 cells	RA (10 μM) + docetaxel (2 nM) for 24 h	ND*	Enhanced docetaxel potency	[70]
Ovarian cancer: SKOV-3/cisplatinresistant cells	RAME* (40 µM) + cisplatin (10 µM) for 24 h	Downregulation of FOXM1-regulated genes	Enhancement of cisplatin potency	[11]
Cervical cancer: HeLa and SiHa cells	RAME* (80 μM) for 24 h	mTOR/S6K1 inhibition	Reversed cisplatin chemoresistance	[66]

RAME*: rosmarinic acid methyl ester; ND*: not determined; IP*: intraperitoneal administration.

## 7. Protective Role of RA against Chemotherapy-Induced Toxicity

This chapter discusses the protective effects of RA against the collateral damage caused by chemotherapy drugs and radiation used in radiotherapy treatments.

### 7.1. RA Prevents Cell Damage Caused by Lipid Peroxidation

Lipid peroxidation is a process where oxidants, such as free radicals or non-radical species, attack lipids containing carbon–carbon double bonds, particularly polyunsaturated fatty acids (PUFAs) found in cell membranes. This process is detrimental to cells because it compromises membrane structure and function [72,73]. During cancer chemotherapy, antineoplastic agents like doxorubicin and 5-FU induce oxidative stress, leading to lipid peroxidation and subsequent tissue damage. For example, doxorubicin contributes to cardiomyocyte loss through apoptosis and dysregulated autophagy triggered by phospholipid oxidation, which can lead to cardiovascular issues [74]. Antimetabolites such as 5-FU generate strong underlying toxicity. clinically. Similarly, 5-FU’s toxicity is associated with the generation of free radicals that enhance lipid peroxidation, contributing to side effects like myelosuppression and cardiotoxicity [75].

Cisplatin, another commonly used chemotherapy drug, induces ototoxicity by generating reactive oxygen species (ROS) in cochlear tissues. The isoform NOX3 of NADPH oxidase is implicated in ROS production in the cochlea, leading to oxidative damage via lipid peroxidation of membrane PUFAs [76]. RA has shown potential in preventing antioxidant activity and scavenging free radicals during chemotherapy-induced lipid oxidation, suggesting a protective role in mitigating oxidative stress-related damage caused by chemotherapy drugs.

The study referenced by [77] demonstrated the antioxidant properties of caffeic acid derivatives, including RA, using both non-cellular and cellular assays. In non-cellular assays, such as the DPPH radical scavenging assay and the Rancimat assay for lipid oxidation prevention, RA and other derivatives exhibited significant antioxidant activity. Specifically, RA showed superior free radical scavenging activity compared to other derivatives, α-tocopherol (vitamin E), and butylated hydroxytoluene (BHT) in corn oil. This indicates RA’s potential efficacy in preventing lipid peroxidation in food oils (Table 2) [77].

In cellular assays using 1,2-dilinoleoyl-sn-glycero-3-phosphocholine (DLPC)/RA liposomes, it was found that RA molecules effectively inserted into lipid membranes and were capable of preventing lipid peroxidation at a molar concentration of 1% without altering membrane structure. This suggests RA’s ability to protect cell membranes from oxidative damage, which is crucial in the context of preventing oxidative stress-related cellular injury [78].

### 7.2. RA in Chemoprotection of Doxorubicin-Induced Toxicity

Doxorubicin (DOX) is the most widely used of these drugs, intercalating with DNA base pairs, and targeting multiple molecular targets to produce a variety of cytotoxic effects such as apoptosis and necrosis in healthy tissue [79]. The heart is the organ most affected by DOX-induced toxicity. However, it also affects other organs such as the brain, kidneys, and liver [80]. DOX-induced cardiotoxicity occurs due to multiple factors, such as increased production of reactive oxygen species (ROS), the release of inflammatory mediators, and disruption of intracellular calcium cycle homeostasis in the heart, which eventually triggers apoptotic signaling pathways and leads to the loss of cardiomyocytes. The massive loss of contractile cardiomyocytes contributes to systolic dysfunction, and their replacement by a non-contractile and poorly conductive collagen scar further impairs the mechanical and electrical functions of the heart [81].

RA has been shown to reduce DOX-induced apoptosis in H9c2 cardiac muscle cells, and reduce intracellular ROS generation through downregulation of c-Jun N-terminal kinase (JNK) and extracellular signal-regulated kinase (ERK), as well as to restore the membrane potential of mitochondria (Δψ) [82]. RA has also shown an antioxidant role, which is evidenced by the ability and recovery of levels of glutathione (GSH), hydrogen peroxide (H_2_O_2_), and superoxide radicals (O_2_·), reducing the expression of malondialdehyde and regulating the expression of antioxidant enzymes such as superoxide dismutase (SOD), as well as upregulating catalase heme oxygenase-1, resulting in significantly improved viability [78].

The cardioprotective effect of RA was investigated in vivo by Zhang et al., who induced DOX-induced cardiotoxicity in male C57/B6 mice through a single intraperitoneal (IP) injection of DOX at a concentration of 15 mg/kg daily. Following injection, mice received oral RA treatment at (100 mg/kg/d) for 7 days. RA administration mitigated cardiomyocyte apoptosis mediated by cardiac fibroblasts. At the molecular level, RA suppressed Fas L expression and release in cardiac fibroblasts, thereby ameliorating DOX-induced cardiotoxicity [81]. In another study, Rahbardar et al. demonstrated the in vivo cardioprotective effect of RA in male Wistar rats. Rats received RA at 40 mg/kg/day intraperitoneally for 4 days prior to DOX treatment (2 mg/kg every 48 h for 12 days). RA treatment attenuated DOX-induced neurotoxicity by exerting antioxidant effects, reducing malondialdehyde levels, and increasing GSH levels [83].

In vitro studies using cardiomyocyte cultures from male Wistar rats demonstrated the anti-lipoperoxidative effects of hydroxycinnamic acids against the DOX–iron complex in rat heart microsomes and mitochondria, with chlorogenic acid exhibiting the highest potency followed by RA and caffeic acid, respectively [84]. Specifically, RA showed superior protection of cardiomyocyte membranes compared to chlorogenic acid. These findings suggest that RA’s antilipoperoxidative effects play a crucial role in its cytoprotective mechanism against DOX-induced injury in vitro. Lipid peroxidation was assessed by measuring malondialdehyde (MDA) levels using thiobarbituric acid (TBA), as MDA is a key indicator of lipid peroxidation [85]. In vivo studies by Rahbardar et al. evaluated MDA levels in cardiac tissue of male Wistar rats pretreated with RA (10 mg/kg/day, IP) for 4 days before DOX injections. The RA-treated group exhibited significantly reduced MDA levels compared to the non-pretreated group [83].

RA is reported to possess antioxidant properties that mitigate oxidative stress in the male reproductive system, akin to taurine, a known amino acid antioxidant that prevents sperm lipid peroxidation induced by DOX treatment across various tissues [86]. Co-administration of RA with DOX has been shown to decrease tissue levels of malondialdehyde (MDA) and increase glutathione peroxidase (GPx) activity, which protects against endogenous hydroperoxide damage. These findings suggest that RA’s antioxidant properties can attenuate testicular tissue damage [87].

Extracts rich in RA have been investigated for their antioxidant properties. Rosemary extracts, known for their high antioxidant activity comparable to BHA (butylhydroxyanisole) and BHT, were studied by Ahmed and Abdella [88]. They found that rosemary extract (125 mg/kg) significantly mitigated doxorubicin-induced toxicity in male albino mice (*Mus musculus*) by inhibiting lipid peroxidation, enhancing cellular antioxidant synthesis, reducing the inflammatory response, and decreasing the apoptotic index.

Additionally, a standardized extract of *Melissa officinalis* (MO), which contains RA as the predominant hydroxycinnamic acid, was tested. In adult male Wistar albino rats treated with a dose of 750 mg/kg/day of MO alongside DOX, significant attenuation of oxidative stress biomarkers in serum was observed, including reduced levels of total oxidants and MDA compared to rats treated with DOX alone. These findings highlight MO’s effectiveness in ameliorating DOX-induced oxidative stress in vivo [89].

To investigate the systemic administration of RA against ototoxicity, male Wistar rats were exposed to intense noise to induce hearing loss, hair cell death, and oxidative stress. They were then treated with RA using two modalities: trans-tympanic (20 μL) and systemic (10 mg/kg) for 3 days before noise exposure. Lipid peroxidation and superoxide production were assessed by immunostaining with dihydroethidium (DHE) and 8-Isoprostane. RA administration effectively mitigated oxidative stress induced by noise exposure, as indicated by reduced superoxide levels. Both trans-tympanic and systemic RA treatments demonstrated comparable protection at both functional and morphological levels against lipid peroxidation [90].

### 7.3. RA in Chemoprotection against Platinum Derivative-Induced Toxicity

Platinum derivatives, including cisplatin, exert their therapeutic effects by damaging cellular DNA, which at high doses induces acute apoptosis through oxidative stress and inflammation [91,92,93]. Cisplatin is widely used in treating solid tumors such as those in the head and neck, breast, ovary, bladder, cervix, and testes, as well as small and non-small cell lung cancers and liver metastases [94,95,96]. Its major dose-limiting side effect is nephrotoxicity, leading to severe renal dysfunction [97]. Cisplatin toxicity involves increased activity of caspase-3, caspase-8, and caspase-9, release of cytochrome c, translocation of apoptosis-inducing factor (AIF), generation of reactive oxygen species (ROS), and activation of NF-κB [98]. Other side effects include ototoxicity, gastrointestinal toxicity, myelosuppression, ovarian toxicity, and neurotoxicity.

RA has been identified as a nephroprotective agent in Wistar rats when administered at a dose of 5 mg/kg/day for two consecutive days following a single intraperitoneal injection of 13 mg/kg cisplatin. RA mitigated histopathological changes and reduced serum creatinine and blood urea nitrogen (BUN) levels. At the molecular level, RA downregulated the expression of CYP2E1 and HO-1 enzymes, thereby decreasing oxidative stress and inflammation by inhibiting NF-κB and TNF-α expression [97].

An additional concern associated with cisplatin use is its potential to cause infertility. It has been observed that 40% of women receiving cisplatin chemotherapy eventually experience ovarian failure, temporary or permanent infertility, and hormonal imbalance [99,100]. Recently, RA has been investigated for its protective effects against cisplatin-induced ovarian damage. RA administration in female Swiss BALB mice subjected to cisplatin treatment increased levels of glutathione (GSH), superoxide dismutase (SOD), catalase, and glutathione peroxidase (GPx) in serum and ovarian tissue. It also decreased levels of pro-inflammatory cytokines such as IL-6, TNF-α, and IL-1β, along with regulating hormonal parameters [101].

Hearing damage is a common consequence of cisplatin chemotherapy, primarily due to the apoptosis of auditory hair cells. Studies on Sprague-Dawley rats (C57BL/6) have shown that RA can inhibit apoptosis in explants of the organ of Corti and HEI-OC1 auditory cells. RA achieves this by inhibiting the downstream signaling pathway of caspase-1 and blocking the activation of NF-κB, thereby mitigating hearing impairment caused by systemic cisplatin chemotherapy [98].

Oxaliplatin, a third-generation platinum derivative, is commonly used as first-line therapy for colorectal, prostate, testicular, and breast cancers, particularly in advanced colorectal cancer combined with 5-FU [102]. Although better tolerated than cisplatin, oxaliplatin-induced peripheral neuropathy (OIPN) remains a significant complication affecting approximately 30–40% of patients, often limiting treatment doses. RA has shown promise as a neuroprotectant against OIPN. In a study on male Sprague-Dawley rats with OIPN induced by oxaliplatin, treatment with RA (50 mg/kg/d for 28 days) demonstrated clinical prevention of functional deficits, allodynia, and cold-induced hyperalgesia. Molecularly, RA preserved mitochondrial function by preventing ATP level depletion induced by oxaliplatin and suppressing inflammatory marker expression [103]. RA’s neuroprotective effects are attributed to the activation of adenosine monophosphate-activated protein kinase (AMPK) in peripheral nerves and the dorsal root ganglion, which negatively regulates glial activation, a key factor in pain progression.

### 7.4. AR in the Prevention of Radiation Therapy Injury

Two studies have explored the potential of RA in radiation protection. The first study investigated RA’s effect on radiation-induced injury to the parotid gland and its mechanisms. Sprague-Dawley rats exposed to 15 Gy of X-rays were treated with various doses of RA (30, 60, and 120 mg/kg) or amifostine (AMI, 250 mg/kg). RA not only reduced apoptosis by inhibiting p53/Jun N-terminal kinase activation but also mitigated parotid gland fibrosis by downregulating inflammatory factors. Compared to AMI, RA demonstrated advantages in long-term efficacy and ease of administration [104].

The second study evaluated RA in combination with other natural acids (caffeic acid, trans-cinnamic acid, p-coumaric acid, and hydroxyphenyl-lactic acid) for radioprotection of skin cells. Human keratinocyte cells (HaCaT) were pretreated with 1 μg/mL of RA before exposure to 4 Gy of γ radiation (1 Gy/min) for 24 h. Intracellular ROS levels induced by radiation were assessed by flow cytometry, and double-stranded DNA damage was evaluated by immunocytochemistry. RA pretreatment conferred 20% protection to HaCaT cells. The study suggests that incorporating RA or these natural acids into chemoradiotherapy regimens could enhance skin radioprotection [105]. Further details of the findings are summarized in Table 3.

## 8. Conclusions

In conclusion, tackling chemoresistance remains a significant clinical challenge, and it is exacerbated by the discrepancy in research emphasis between advancing cancer treatments and managing treatment-related symptoms and toxicities. Rosmarinic acid (RA), a polyphenol found in medicinal plants, demonstrates promising synergistic effects when combined with first-line anticancer drugs, interacting with critical signaling pathways to overcome resistance in cancer cells.

Looking ahead, the extensive adoption of RA in the food industry offers considerable potential for developing functional food products. Leveraging established safety parameters and insights from bioactivity studies can accelerate the integration of RA into innovative food formulations, capitalizing on its demonstrated health-enhancing properties. Incorporating RA or RA-containing extracts can mitigate food ingredient oxidation and extend product shelf life, addressing both microbiological and sensory concerns. This approach not only relieves economic burdens in the food sector but also promises beneficial health effects for consumers.

Moreover, RA shows promise as a chemoprotective agent, mitigating side effects in normal tissues affected by systemic cancer treatments like DOX or platinum derivatives. This is attributed to RA’s anti-inflammatory effects, mediated through modulation of the NF-κB pathway, and its capacity to bolster cellular antioxidant defenses, thereby reducing lipid peroxidation. While RA emerges as a promising adjunct therapy for cancer treatment, further preclinical and clinical investigations, coupled with dosage optimization, are crucial to fully elucidate its therapeutic potential.

## Figures and Tables

**Table 2:** Induction time of lipid oxidation for RA and caffeic acid derivatives [77].

Compound	Inhibition % DPPH	Antioxidant Index in Lard	Antioxidant Index in Corn Oil
Control	-	1.00	1.00
CA	51.5	11.10	2.51
CAPE	57.5	9.86	2.41
RA	85.6	9.76	2.97
CGA	36.3	7.83	2.33
α-tocopherol	32.5	11.10	1.93
Control BHT	8.9	2.77	1.72

CA: caffeic acid; CAPE: caffeic acid phenetyl ester; CGA: chlorogenic acid.

**Table 3:** Signaling pathways and proteins affected by rosmarinic acid in its role as a chemoprotectant against anticancer chemotherapy-induced toxicity.

Signaling Pathway/Proteins	Organs or Tissues	Treatment Conditions	Main Findings	References
Fas/FasL cell signaling pathway	Cardiac fibroblasts Cardiomyocytes	In vivo: DOX* (15 mg/kg), one day before treatment with RA (100 mg/kg/day), orally for 7 days	RA Suppressed FasL Expression and Release, Alleviating Apoptosis in Cardiomyocytes RA attenuated heart and body weight loss in mice	[81]
JNK and ERK activity GSH, SOD and Bcl-2 levels	Cardiac muscle cells	In vitro: RA (20 mg/mL), for 30 min prior to treatment with DOX (1, 2 or 4 μM)	RA inhibited JNK and ERK activation in H9c2 RA improved viability of H9c2 cells by preventing DOX-induced caspase 3 activation RA positively regulated GSH, SOD, reduced intracellular ROS generation, and restored mitochondrial membrane potential (Δψ)	[82]
MDA and GSH levels	Heart	In vivo: RA (40 mg/kg/day), IP for 16 days prior DOX treatment (2 mg/kg/48 h), IP for 12 days, starting on the 4th day	RA reversed the ECG abnormalities and reduction in systolic and diastolic pressure caused by DOX RA decreased heart weight and improved DOX-induced histopathology RA attenuated MDA levels and increased GSH levels in cardiac tissue	[83]
CYP2E1 and HO-1 NF-κB pathway	Kidney	In vivo: cisplatin (13 mg/kg), in a single-dose IP for 2 days before RA (5 mg/kg/day), orally for 2 days	RA downregulated elevated CYP2E1 and HO-1 levels, and reduced oxidative stress caused by cisplatin. RA decreased proinflammatory proteins NF-κB and TNF-α, and the expression of p53 and caspase-3 in the kidneys	[97]
GSH, SOD, catalase and GPx IL-6, TNF-α, IL-1β	Ovarian tissue	In vivo: cisplatin (7 mg/kg), IP on day 1 prior RA (10 mg/kg) for 14 days	RA reduced cisplatin-induced oxidative stress RA decreased the levels of IL-6, TNF-α, IL-1β, inflammatory, and apoptosis parameters in a dose-dependent manner (*p* < 0.001)	[101]
Caspase signaling pathways NF-κB pathway	Aauditory hair cells Cochlea explants	In vitro: RA (100 µM) for 4 h prior to cisplatin (20 µM) for 48 h. In vivo: RA (4 mg/kg/day), for 4 days before cisplatin treatment	RA blocked caspases 3 and 9 activation, cytochrome c release, and ROS generation induced by cisplatin in HEI-OC1 cells RA inhibited cisplatin-induced NF-κB activation and its DNA binding activity	[98]
Mitochondrial function AMPK status Inflammatory Markers	Neuronal cells Peripheral nerve	In vitro: RA (50 μmol) plus oxaliplatin (50 μmol) for 24 h In vivo: Oxaliplatin (4 mg/kg), IP twice a week for 4 weeks prior RA (50 mg/kg/day), orally for 28 days	RA improved mitochondrial function and prevented oxaliplatin-induced loss of ATP levels in Neuro-2a (N2a) cell line RA inhibited the activation of spinal glial cells and suppressed the expression of inflammatory markers TNF-α and IL-6 in spinal glial cells RA contributed to neuroprotective activity through AMPK activation in peripheral nerves and dorsal root ganglion	[103]
Cellular ROS	Skin cells	In vitro: RA (1 µg/mL) for 24 h prior irradiation at 2, 4, and 8 Gy (1 Gy/min)	RA decreased radiation-induced ROS with RA by 21% compared to control Pretreatment with RA increased cell survival by approximately 20% at a level of 8 Gy	[105]

DOX*: doxorubicin.
